# Response of microRNAs to cold treatment in the young spikes of common wheat

**DOI:** 10.1186/s12864-017-3556-2

**Published:** 2017-02-28

**Authors:** Guoqi Song, Rongzhi Zhang, Shujuan Zhang, Yulian Li, Jie Gao, Xiaodong Han, Mingli Chen, Jiao Wang, Wei Li, Genying Li

**Affiliations:** 1 Crop Research Institute, Shandong Academy of Agricultural Sciences, Jinan, 250100 China; 2 Key Laboratory of Wheat Biology & Genetic Improvement on North Yellow & Huai River Valley, Ministry of Agriculture, Jinan, 250100 China; 3 National Engineering Laboratory for Wheat & Maize, Jinan, 250100 China

**Keywords:** Cold stress, Degradome, Inflorescence development, MicroRNA, Wheat

## Abstract

**Background:**

MicroRNAs (miRNAs) are a class of small non-coding RNAs that play important roles in biotic and abiotic stresses by regulating their target genes. For common wheat, spring frost damage frequently occurs, especially when low temperature coincides with plants at early floral organ differentiation, which may result in significant yield loss. Up to date, the role of miRNAs in wheat response to frost stress is not well understood.

**Results:**

We report here the sequencing of small RNA transcriptomes from the young spikes that were treated with cold stress and the comparative analysis with those of the control. A total of 192 conserved miRNAs from 105 families and nine novel miRNAs were identified. Among them, 34 conserved and five novel miRNAs were differentially expressed between the cold-stressed samples and the controls. The expression patterns of 18 miRNAs were further validated by quantitative real time polymerase chain reaction (qRT-PCR). Moreover, nearly half of the miRNAs were cross inducible by biotic and abiotic stresses when compared with previously published work. Target genes were predicted and validated by degradome sequencing. Gene Ontology (GO) enrichment analysis showed that the target genes of differentially expressed miRNAs were enriched for response to the stimulus, regulation of transcription, and ion transport functions. Since many targets of differentially expressed miRNAs were transcription factors that are associated with floral development such as ARF, SPB (Squamosa Promoter Binding like protein), MADS-box (MCM1, AG, DEFA and SRF), MYB, SPX (SYG1, Pho81 and XPR1), TCP (TEOSINTE BRANCHED, Cycloidea and PCF), and PPR (PentatricoPeptide Repeat) genes, cold-altered miRNA expression may cause abnormal reproductive organ development.

**Conclusion:**

Analysis of small RNA transcriptomes and their target genes provide new insight into miRNA regulation in developing wheat inflorescences under cold stress. MiRNAs provide another layer of gene regulation in cold stress response that can be genetically manipulated to reduce yield loss in wheat.

**Electronic supplementary material:**

The online version of this article (doi:10.1186/s12864-017-3556-2) contains supplementary material, which is available to authorized users.

## Background

Low temperature is one of the most important environmental stimuli that affect plant growth and development. Common wheat, as a classical vernalization-required plant, is tolerant to lower temperatures at the vegetative development stage. However, it will suffer frost injury when low temperatures coincide with the reproductive stage of wheat. In spring, the low temperature occurring at the stage when pistil and stamen differentiate into anthers will cause anther sterility and floret abortion, leading to significant yield loss in wheat [1]. In the vegetative developmental stage, vernalization genes *VRN1*, *VRN2*, and *VRN3* regulate the transition between the vegetative and reproductive stages and interconnect with the cold acclimation regulation locus *FR2* including the *CBF* and *COR* genes. These genes have been characterized to respond to freezing tolerance [2, 3]. For the spring freeze, stress-related, photosynthesis, and plastid-associated genes are demonstrated as responsive genes by transcriptomic and proteomic analyses [4]. For example, a set of spring freeze-responsive genes were identified by using the Affymetrix GeneChip in barley [5], while using a similar approach a variety of genes are reported to be involved in wheat spring freeze stimulus including WCOR413, LEA, glycine-rich RNA-binding protein, ferritin, aquaporin 2, and a pathogen-induced protein, the ice recrystallization protein, cold-related proteins, CBF transcription factors, calcium-dependent protein kinases, Na+/H+ antiporters, aquaporins, and many metabolic enzymes [6]. Recently, RNA-seq and digital gene expression analysis also identified a series of protein-coding genes from cold-treated young spikes in wheat [7]. In general, frost resistances at the vegetative stage and the reproductive stage seem to confer overlapping regulatory networks such as the one mediated by the *CBF* gene. Despite this, gene networks for frost resistance at the reproductive stage appear to be more complicated and involve more physiological processes.

MicroRNAs (miRNAs) are classical small non-coding RNAs that guide post-transcriptional gene regulation. MiRNAs have been shown to play key roles in various biological processes, including development, hormone responses and stress adaptation [8–11]. Recently, many studies have demonstrated the role of plant miRNAs in cold stress response. In *Arabidopsis*, 16 miRNAs, including miR156, miR159, miR164, miR165, miR168, miR169, miR172, miR319, miR389, miR393, miR396, miR397, miR398, miR400, miR402, and miR408, were identified by RNA gel blot analysis [12], microarray analysis [13], and a computation-based approach to be related to cold response [14]. In *Populus*, 19 cold stress-responsive miRNAs were identified by miRNA microarray [15], among which miR156, miR164, miR168, miR169, miR393, and miR396 were overlapped with those in *Arabidopsis*. In addition, 25 species-specific miRNAs were identified as cold regulated in *Brachypodium* by high-throughput sequencing [16]. These works indicate conserved mechanisms for cold-responsive miRNAs as well as the diverged species-specific regulation. In common wheat (*Triticum aestivum*), many conserved, *Triticum*-specific, and wheat-specific miRNAs have been identified, including the wheat A genome progenitor, the wheat D genome progenitor, and the AABBDD hexaploid wheat [17–26]. MiRNAs have been identified to play important roles in various stresses in wheat [9], including abiotic stresses such as salt [27, 28], drought [28, 29], dehydration [30], phosphorus [31], heat [32] and biotic stresses such as the fungal infection of powdery mildew [32] and *Puccinia striiformis f. sp. Tritici* [33]. So far, six miRNAs, miR167c, tae-miR167d, tae-miR172a, tae-miR393, tae-miR396a, and tae-miR444c.1 were considered to be cold responsive in a thermosensitive genic male sterile (TGMS) lines of wheat [34]. In addition, seven miRNAs, miR159, miR164, miR169, miR319, miR398, miR1029, and miR1126 were also identified to be cold-responsive in the seedling of wheat [9].

Despite these efforts, our knowledge about the roles of miRNAs in wheat response to cold stress is still limited. Here, we sequenced small RNAs of cold treated young spikes at the pistil and stamen differentiation stage and controls. A diverse set of wheat small RNAs were identified, some of which were differentially expressed between cold-treated and control samples. MiRNA targets were then validated by degradome sequencing. By comparing with miRNAs reported previously under other stress and developmental conditions, we provided a comprehensive picture of miRNA functions in cold treated wheat inflorescences that may assist cold tolerance improvement in wheat.

## Methods

### Plant materials

The seeds of common wheat cultivar JM22 were germinated and grown in a 4 °C chamber for vernalization. After 40 days, the seedlings were transplanted to an artificial climate chamber with a relative humidity of 75% and 22 °C/16 °C day/night temperatures with a light intensity of 3000 lx. Approximately 20 days after transplanting, most of the young spikes progressed into the pistil and stamen differentiation stage, and then the seedlings were divided into two groups. One group was moved to a chamber and grew under cold treatment at 0 °C for 48 h, while the other was used as the control sample and remained in the 22 °C/16 °C growth chamber.

### RNA isolation and small RNA and degradome library construction

Young spikes were pooled for the control samples or cold treatment samples after cold treatment for 48 h at 0 °C with two replications, respectively, and then were stored in liquid nitrogen for RNA extraction. Total RNA was isolated using the TRIzol reagent according to the manufacturer’s instructions (Invitrogen, Carlsbad, CA, USA). RNA quality was tested using a 2100 Bioanalyzer RNA Nanochip (Agilent, Santa Clara, CA, USA). The RNA concentration was quantified using a NanoDrop ND-1000 Spectrophotometer (Nano-Drop, Wilmington, DE, USA). Two degradome libraries for the control and cold treatment samples were constructed following the method by German et al [35].

### Bioinformatics analysis of small RNA sequencing data

Small RNA and degradome reads were generated from Illumina HiSeqTM analysis. Using the Fastx-toolkit pipeline (http://hannonlab.cshl.edu/fastx_toolkit/), the raw sequencing data were pre-processed to remove low-quality reads, reads smaller than 18 nucleotides (nt), trim adaptor sequences, and contamination formed by adaptor-adaptor ligation. Regarding the small RNA sequencing data, clean reads ranging from 18 to 30 nt were aligned to the Chinese Spring genome from the IWGSC (version 2) website and the transcriptome dataset for young spikes of the common wheat downloaded from the GEO dataset as SRX375489 by the bowtie program [7]. The reads were categorized as the exon, intron, and repeat-associated small RNAs with a perfect match to the reference genome. Perfectly matching sequences were identified for further analysis. The reads were annotated as rRNA, tRNA, small nuclear RNA (snRNA), and small nucleolar RNA (snoRNA) by alignments against the Rfam 12.0 database (http://rfam.xfam.org/). The reads were identified as known miRNAs by the BLAST search against the miRBase 21 database with at most two mismatches (http://www.mirbase.org/), and the wheat microRNAs from PNRD (http://structuralbiology.cau.edu.cn/PNRD), PmiRExAt (http://pmirexat.nabi.res.in/), and IWGSC miRNA dataset [36] with the perfect match. After the removal of the known small RNAs and repeat-associated small RNAs, we used the miRdeep-P program with default parameters to predict the novel miRNAs using a plant-specific scoring system and filtering criteria based on a probabilistic model of miRNA biogenesis [37].

### Bioinformatics analysis of degradome sequencing data

For the degradome sequencing data, the clean reads were generated using the same method for the small RNA sequencing analysis. Next, the clean reads were used to identify the cleavage sites of the miRNA-target interaction by the program CleaveLand [38]. The transcripts included the CDS, UTR of Chinese Spring (version 2.2) downloaded from the IWGSC website, and the assembled transcriptome from the Zhang et al downloaded from the GEO dataset as SRX375489 [7]. The cleavage sites were classified as the 0, 1, 2, 3, and 4 categories according to the abundance of reads in the slice sites along the whole transcripts with a *P-value* less than 0.05.

### Quantification of miRNA expression level by qRT-PCR

qRT-PCR was performed to determine the validity of miRNA by deep sequencing for expression profile analysis. Total RNA was extracted as described above. According to previous reports [20, 39], the miRNA abundance was detected. Briefly, we first polyadenylated the total RNA (3 μg) including miRNAs, and then used the Mir-X^TM^ miRNA First-Strand Synthesis kit (Clontech, Inc., Terra Bella, USA) to reverse transcribe poly (T) adapters into cDNA. Using the internal reference gene *UBQ*, we normalized the cDNA products [20]. These products were used as templates for qRT-PCR. qRT-PCR was performed on a LightCycler® 480 (Roche, Switzerland) using LightCycler 480 SYBR Green I (Roche, Switzerland). Along the entire miRNA sequence and adapter sequence provided by miRNA First-Strand synthesis kit (Clontech, Inc., Terra Bella, USA), the miRNA-specific forward primer for each miRNA and the universal reverse primer were designed. The primers were shown in the Additional file 1: Table S1. qPCR was performed according to the following protocol: 95 °C for 2 min, 40 cycles of 95 °C for 5 s and 60 °C for 20 s, followed by a thermal denaturing step to generate the melt curves. All of the reactions were performed in triplicate, including non-template controls. Statistical analysis was performed using the 2^-ΔΔct^ method [40].

### Quantification of miRNA targets by qRT-PCR

Total RNA was extracted from samples according to the manufacturer’s instructions of the RNAiso plus kit (TaKaRa, Dalian, China). They were reverse transcribed with poly (T) adapters into cDNA using a PrimeScript^TM^ RT reagent Kit with gDNA Eraser kit (TaKaRa, Dalian, China). The cDNA products of each tissue were normalized using *Actin* (AB181991) as the internal reference gene [7]. These products were used as templates for qRT-PCR. qRT-PCR was performed on a LightCycler® 480 (Roche, Switzerland) using LightCycler 480 SYBR Green I (Roche, Switzerland). The qRT-PCR programmes were performed according to the following protocol: 95 °C for 2 min, 40 cycles of 95 °C for 5 s and 56 °C for 20 s, according to a thermal denaturing step to generate the melt curves. All reactions were run in triplicates, including the non-template controls. Statistical analysis was performed using the 2-ΔΔct method [40]. The primers were shown in the Additional file 1: Table S1.

## Results

### Sample collection and small RNA sequencing

**Fig. 1:**
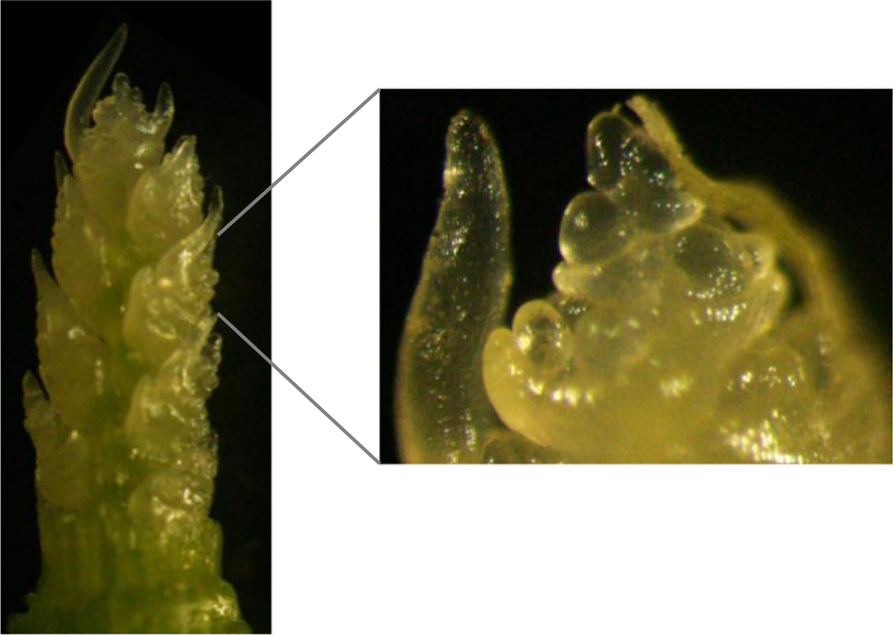
Young spikes at the pistil and stamen differentiation stage

**Fig. 2:**
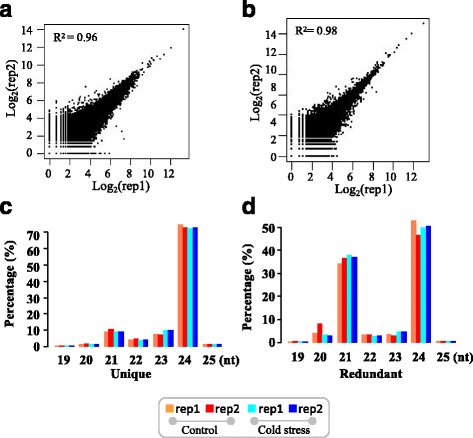
**a-b** Correlation of the small RNA libraries of the control samples between the two replicates (**a**) and cold-stressed samples between the two replicates (**b**) in young spikes at the differentiation stage of pistil and stamen. **c-d** Length distribution of distinct (**c**) and abundant (**d**) small RNAs mapped on the Chinese Spring genome. Red, coral red, cyan and blue colours represent the control samples with two replicates and cold-stressed libraries with two replicates in young spikes at the differentiation stage of pistil and stamen

To investigate the response of miRNAs to cold stress, four libraries of small RNAs were constructed from young spikes at the pistil and stamen differentiation stage with and without cold stress, respectively (Fig. 1), and with two biological replications. After the removal of contaminating reads, 20–22 million clean data representing 6.4–8.0 million distinct sequences were obtained (Table 1). The high correlation between the two replicates, with R^2^ = 0.96 for the control samples and R^2^ = 0.98 for the cold-stress samples, indicated the high quality of the small RNA libraries (Fig. 2a & b). Next, we mapped these sequences to the genome of Chinese Spring. Approximately 81–82% of the small RNAs could be perfectly mapped to the genome, and there was no bias distribution among the three sets of subgenomes A, B, and D (Table 1 & Additional file 1: Table S2). Most of the small RNAs were located in the intergenic and repeat element regions, while only 3 to 6% small RNAs were mapped to exon and intron regions. Intriguingly, in the exon regions, the small RNAs located in the subgenome B were significantly lower than those in the other two subgenomes, A and D (Additional file 1: Table S2 & Additional file 2: Figure S1), suggesting possible subgenome dominance of small RNA regulation functions. The lengths of the small RNAs ranged from 14 to 43 nucleotides (nts). Most of the small RNAs ranged from 20 to 24 nt and were used for subsequent analysis. For distinct small RNAs, the 24-nt category was the most abundant, representing 71–73% among unique reads and 45–53% for redundant ones, followed by 21-nt small RNAs (Fig. 2c & d). As shown in other plants, 24-nt siRNAs may play a role in maintaining genome integrity and stabilization by heterochromatin formation [41].

**Table 1:** Small RNA mapping information using the Chinese Spring genome as a reference^a^

Mapping to genome	Control		Cold treated	
	Replicate 1	Replicate 2	Replicate 1	Replicate 2
Total reads	22597554	20332674	22083486	22660147
Genome	18463571 (81.71%)	16773198 (82.49%)	17963869 (81.35%)	18516384 (81.71%)
Intron	813339 (3.60%)	698142 (3.43%)	781581 (3.54%)	865990 (3.82%)
Exon	823689 (3.65%)	1355522 (6.67%)	672555 (3.05%)	651084 (2.87%)
Repeat	6137517 (27.16%)	5819410 (28.62%)	5964382 (27.01%)	6221074 (27.45%)
rRNA	96048 (0.43%)	98947 (0.49%)	99067 (0.45%)	86400 (0.38%)
tRNA	662619 (2.93%)	1250548 (6.15%)	496776 (2.25%)	464297 (2.05%)
snoRNA	3600 (0.02%)	3373 (0.02%)	3650 (0.02%)	3489 (0.02%)
snRNA	8789 (0.04%)	8726 (0.04%)	8371 (0.04%)	9541 (0.04%)

^a^Chinese genome version 2

### Identification of miRNAs in wheat young spikes

The functions of many miRNAs are conserved in plants, and these miRNAs play important roles in development and stress responses [8]. MiRNAs were generated across the evolution of plants, the miRNAs with important functions were retained as the plant-conserved miRNAs, and some miRNAs as monocot-specific or eudicot-specific miRNAs came into being after the divergence of monocot and eudicot, and some miRNAs as species-specific miRNAs were generated after the species divergence [42]. To identify the plant-conserved miRNAs in wheat, we aligned the cleaned small RNAs to known plant miRNAs downloaded from the miRBase (release 21.0), PNRD, PmiRExAt, and IWGSC miRNA database using the BLASTN program. In total, 192 known miRNAs from 105 families were obtained. Among them, 25 conserved miRNA families covered almost all of the plant-conserved miRNA families. Twenty-four grass-specific miRNA families were identified, including those in rice, maize, sorghum, and *Brachypodium*. Recently, using the next-generation sequencing, many miRNAs were identified in the bread wheat, their progenitor *T. Urartu* and *A. tauschii*, emmer wheat *T. turgidum*, and barley [43]. These miRNAs were defined as *Triticum*-specific miRNAs. Here, 13 *Triticum*-specific miRNAs, as well as 43 wheat-specific miRNAs, were identified (Additional file 1: Table S3). After the removal of these known miRNAs and Rfam-associated small RNAs, we aligned the remaining small RNAs to the genome for novel miRNA discovery. To date, many wheat-specific miRNAs have been deposited in the miRBase across different development stages and different stress responses. However, based on the special feature of the canonical stem-loop regions of longer RNA precursors, we identified additional nine novel miRNAs in the four small RNA samples that met the previous reported criteria by the miRDeep-P program [37]. Seven of them presented the miRNA* sequences indicating bona fide miRNAs (Additional file 1: Table S4). Five of them were 24 nt in length, and four had 5’ terminal adenine nucleotides. The adenine preferences in the 5’ terminus were processed by the AGO4 protein. Thus, these newly discovered novel miRNAs should increase the number of known miRNA families.

### The abundance of miRNAs in wheat young spikes

To predict the potential roles of novel miRNAs, we investigated their expression profiles. We found that 14 miRNAs from nine families had reads more than ten thousands in at least one sample. Most highly abundant miRNAs were conserved miRNAs such as miR168, followed by miR166, miR167, and miR172, which also highly expressed in other plants [34, 44]. Intriguingly, grass-specific miR5062, *Triticum*-specific miRNAs including tae-miR9672 and miR9863, as well as wheat-specific tae-miR9663 and miR5168, also showed quite high expression levels (Additional file 1: Table S3). There were 24 miRNAs from 16 families with more than one thousand reads in at least one sample. They were four plant conserved miRNAs, four grass-specific miRNAs, eight *Triticum*-specific miRNAs and four wheat-specific miRNAs. The high expression levels of these miRNAs may suggest their important biology functions. By contrast, some miRNA families, including plant-conserved miRNAs, were expressed at a lower abundance (Additional file 1: Table S3). For the novel miRNAs, miR1002 and miR1003 were intermediately expressed with reads of more than one thousand, while the other miRNAs were lowly expressed with only about one hundred reads (Additional file 1: Table S4). Thus, miRNAs were transcribed at various levels in young spikes during the differentiation of the pistil and stamen in wheat.

### Differential expression of miRNAs under cold treatment

**Fig. 3:**
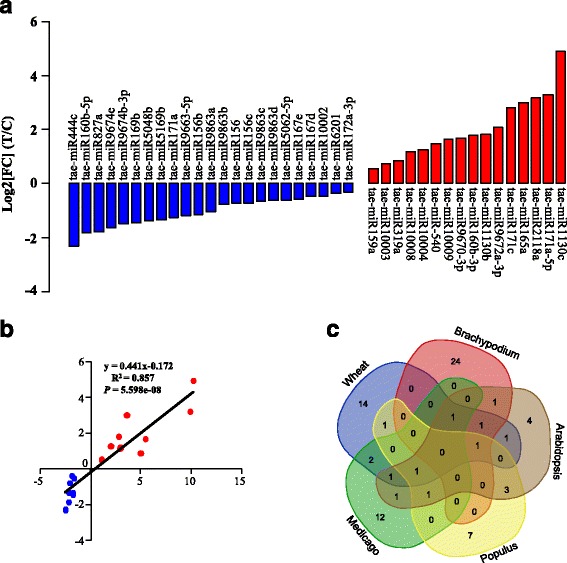
Differential expression profiles of miRNAs. **a** Fold change of differentially expressed miRNAs between the control and cold-stressed libraries in young spikes at the pistil and stamen differentiation stage. Red and blue colours represent up-regulated and down-regulated expression. **b** Correlation of the differentially expressed miRNAs validated by qRT-PCR compared to the Illumina sequencing. **c** Venn diagram of the microRNAs response to cold stress among wheat, *Brachypodium*, *Medicago*, *Populus*, and *Arabidopsis*

As have been shown in previous studies, miRNAs are often associated with cold response in many plants such as *Arabidopsis*, *Populus*, *Brachypodium*, and *Medicago* [45]. Here, we performed differential expression analysis of miRNAs in young spikes with and without cold treatment. After normalizing reads of each miRNA as ‘transcripts per million’ (TPM) for each sample, we characterized 39 differentially expressed miRNAs (FDR < 0.001) from 28 miRNA families using the DEGseq program (Fig. 3a) [46]. Twelve of these differentially expressed miRNA families were conserved in plants, two were grass-specific miRNAs, six *Triticum*-specific, three wheat-specific miRNAs, and five were newly identified novel miRNAs. Among them, 23 were down-regulated, and 16 were up-regulated with differential expression fold changes ranging from 1.2 to 30 times (Fig. 3a).

To confirm the sequencing data, we selected 18 differentially expressed miRNAs for qRT-PCR detection. The expression means of cold responsive miRNAs as detected as by qRT-PCR was very similar to the fold change of the sequencing reads with a high correlation coefficients (R^2^ = 0.832; *P-value* = 5.344e-08) (Fig. 3b). For example, miR156, miR169, miR171a, miR444c, miR827, and miR5048a were identified by sequencing as down-regulated. Such patterns were confirmed by qRT-PCR. Similarly, miR159a, miR160b, miR165a, miR171c, miR319a, miR2118a, miR1130c, and the novel miRNAs miR10008, miR10004, and miR10009 were also validated as being up-regulated by qRT-PCR, consistent with the results of Illumina sequencing (Additional file 1: Table S5). These data demonstrated the effectiveness and accuracy of high-throughput sequencing.

Among the 23 differentially expressed known miRNA families, nine were overlapped with cold-stress miRNAs from other species, such as *Arabidopsis* [12–14], *Medicago* [45], *Populus* [15], and *Brachypodium* [16] (Fig. 3c). MiR169, miR172, miR156, miR319, miR159, and miR396 showed the cold-stress response in at least three species, and miR160, miR165, miR167, and miR171 overlapped with *Populus, Arabidopsis*, and *Medicago* (Additional file 1: Table S6). However, some miRNAs were found to be species-specific (Additional file 1: Table S6), suggesting divergent regulatory pathways in wheat.

### MiRNA target prediction

To understand miRNA functions, we performed target prediction of miRNAs by the TargetFinder program [47] and targets with scores less than four were further analysed. We found that targets of many known miRNAs were mostly associated with development and stress (Additional file 1: Table S7). For example, miR156 targets an *SPB* gene while miR172 targets an *AP2-like ethylene-responsive transcription factor* (*AP2*), both of which mediate the transition of the vegetative stage to reproductive stage [48–50]. Other cold responsive miRNAs, MiR319 and miR444, targeted *TCP* and *MADS-box* genes were involved flower development [51, 52]. Additionally, miR390, miR5071, miR2118, miR9863 and miR7757 were predicted to target the *Leucine-rich Repeat Receptor-like protein kinase* family (*LRR*) that are involved in disease resistance [53, 54], as well as miR1120, miR1127, miR1130, miR1137, miR1439, miR5049, miR5062, and miR9673 that regulated the *WD* domain gene for flower development and the immune system [55–57] (Additional file 1: Table S7).

For novel miRNAs, a total of eight miRNA-target interaction were predicted. As shown in Additional file 1: Table S8, the predicted targets include gene encoding zipper proteins, binding proteins, protein kinases, enzymes and transporters that are potentially involved in multiple cellular processes. Some target genes have been shown to be also involved in stress responses. For instances, the homeobox-leucine zipper protein family regulated by tae-miR1002 and tae-miR1007 has been shown to be involved in the water stress and abscisic acid treatment in *Arabidopsis* [58], while the CASP-like protein, which is targeted by tae-miR1005, mediates the environmental stress response in plants except for the pathogen-induced membrane protein gene in bacterial disease resistance and oomycete disease susceptibility in pepper [59, 60]. In addition, the LRR, which participates in plant defence, regulated of the developmental process and the sensing or transduction of hormone signals in plants [61]. These genes were predicted to be regulated by tae-miR1004 and tae-miR1005. Also, the sugar transporter gene that was targeted by the tae-miR1007 plays a central role in pathogen resistance in rice and cassava [62].

To confirm the coordinated expressions of target genes with those of cold responsive miRNAs, 12 predicted target genes were selected to perform qRT-PCR assays. The results showed that six target genes displayed coordinated expression patterns with corresponding miRNAs while other six showed expression patterns opposite to the expected (Additional file 1: Table S5), which could be caused by more complicated regulation of the target genes such as by other transcription factors.

### MiRNA target validation by degradome sequencing

**Fig. 4:**
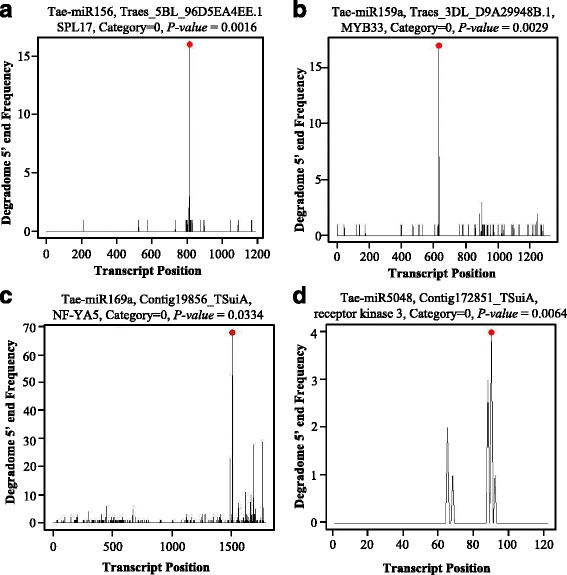
Target plots (T-plots) of miR156 (**a**), miR159 (**b**), miR169 (**c**), and miR5028 (**d**) characterized by degradome sequencing. The abundance of the signature tags was plotted along the indicated transcript. The red dots indicate the predicted cleavage sites on the x axis; the black line indicates the signatures produced by miRNA-directed cleavage

To validate that miRNAs found there can indeed cleave predicted targets, we performed degradome sequencing using the same wheat inflorescence samples. After removing contaminating reads, 18,427,417 and 20,253,810 clean reads were obtained in the two degradome libraries, respectively, one control and one cold treated. 99.3% and 99.4% of them were 20 and 21 nts in length, respectively (Additional file 2: Figure S2a). A total of 23,253,400 reads (60.12%) were overlapped between the two libraries. Among them, 15,879,947 (86.17%) and 17,063,879 (84.25%) reads were aligned to the CDS sequences comprising the Chinese Spring and transcriptome datasets, respectively [7]. Using the CleaveLand program, 109 known miRNAs from 62 families and 112 from 65 families were confirmed to cleave the targets with a *P-value* less than 0.05 using the degradome sequences in the control and cold-stressed libraries, respectively (Additional file 1: Table S9). One hundred and twenty two miRNAs from 70 miRNA families were verified to cleave the targets using the degradome sequences in at least one library. Totally, 99 miRNAs from 57 families were demonstrated to interact with targets in the two libraries. In total, 457 and 484 transcript cleaved sites were confirmed in degradome libraries, and more than half of them were either defined as the ‘0 Category’ with the maximum tags or only one peak at the cleavage site on the transcript (Additional file 2: Figure S2b). Examples for the ‘0 Categories’ are shown in Fig. 4a-d. In addition, 312 miRNA-transcript pairs were shared between the two libraries, and 145 and 172 were characterized specifically in the control and cold stress libraries, respectively. Among transcripts detected by degradome sequencing, 344 transcripts were predicted as targets with a score less than four using the TargetFinder program. For the novel miRNAs, all nine miRNAs were identified to regulate the 32 transcripts in the two libraries. There were six miRNAs with 20 interaction transcripts overlapped between the two libraries, in which three miRNAs with three interaction transcripts specifically were identified in the control libraries and four with nine targeting transcripts were specifically identified in the cold stress libraries (Additional file 1: Table S10). Moreover, as shown in Table 2, a significant number of transcripts detected by degradome sequencing have been reported previously such as miR156-*SBP*, miR172-*AP2*, miR160-*ARF* (*Auxin Response Factor*), miR169-NFYA (*Nuclear transcription Factor Y subunit A*), miR319-*TCP*, and MiR9863-*NBS*, indicating conserved miRNA-target interaction in plants.

**Table 2:** MiRNA targets validated by degradome sequencing^a^

miRNA Classification	miRNA families	Target verified by the Degradome tags
Conserved in plants	miR156	Squamosa promoter-binding-like protein (SPB)
	miR159	myb domain protein (MYB)
		Disease resistance protein
	miR160	auxin response factor (ARF)
	miR166	Homeobox-leucine zipper protein HOX9 (START domain)
	miR167	Auxin response factor (ARF)
	miR168	Argonaute family protein (AGO)
	miR169	Nuclear transcription factor Y subunit A (NTFYA)
	miR172	AP2-like ethylene-responsive transcription factor (AP2)
	miR2118	Emsy N Terminus (ENT)/plant Tudor-like domains-containing protein (EMSY N-terminal)
	miR2275	Cathepsin B-like cysteine proteinase
	miR3057	subtilisin-like serine protease 2 LENGTH = 764
	miR3062	Mitochondrial transcription termination factor family protein LENGTH = 390
	miR3084	Disease resistance protein
	miR319	TCP family transcription factor (TCP)
	miR393	Transport inhibitor response 1-like protein
	miR394	F-box family protein (F-box)
	miR396	Growth-regulating factor (GRF)
Grass-specific	miR1122	fructose-bisphosphate aldolase 2 LENGTH = 398
	miR444	Regulator of chromosome condensation (RCC1) family
		RING finger protein 5
	miR5048	receptor kinase 3 LENGTH = 850
	miR5049	Pre-mRNA-processing-splicing factor LENGTH = 2359
	miR5062	Ubiquitin carboxyl-terminal hydrolase
		Argonaute family protein (AGO)
		Elongation factor 1-beta
	miR5071	Disease resistance protein
*Triticum*-specific	miR5168	Homeobox-leucine zipper protein (START domain)
	miR5048	receptor kinase 3
	miR9674	Pentatricopeptide repeat (PPR)
	miR9772	UPI000234FD81 related cluster
	miR9863	Disease resistance protein
Wheat-specific	miR9662	Mitochondrial transcription termination factor family protein
	miR9676	alpha/beta-Hydrolases superfamily protein
	miR9679	Elongation factor 1-gamma

^a^At least two transcripts were present in the degradome data

### Gene enrichment (GO) analysis for target genes of differentially expressed miRNAs

**Fig. 5:**
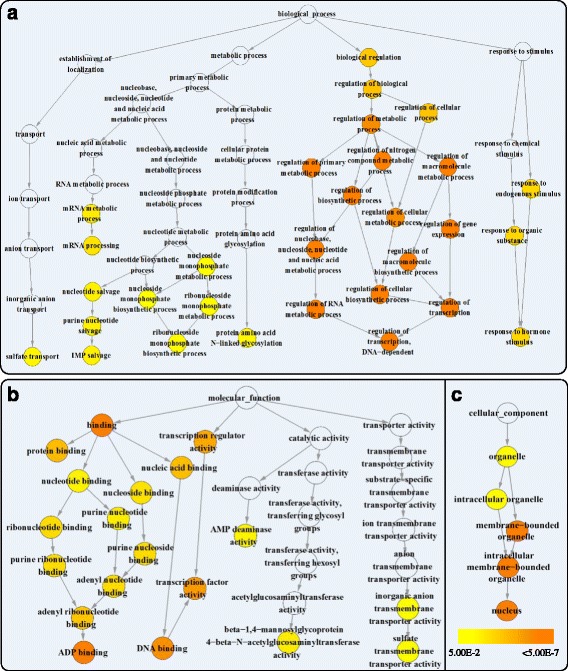
GO enrichment analysis for the targets of significantly differentially expressed miRNAs. Cytoscape views of enriched GO terms were shown in biological process (**a**), molecular function (**b**), and cellular component (**c**) for the targets of differentially expressed miRNAs by cold stress. The coloured nodes and white nodes represent the GO terms enriched and non-enriched in the target genes, respectively

To better understand the function of these differentially expressed miRNAs, enrichment analysis for 685 target genes of significantly differentially expressed miRNAs was analysed using the BINGO program [63]. The GO term annotation of wheat genes was performed according to the Chinese Spring genome release available from the IWGSC website. The results showed that 30, 19 and 5 GO terms were significantly enriched (FDR < 0.05) in the biological process, molecular function, and cellular component categories, respectively. For the biological process, most of the enriched GO terms were classified as the biological regulation such as regulation of transcription, RNA metabolic process, and macromolecule biosynthesis process, suggesting that changes in gene transcription regulation occur in response to the cold stimulus in wheat. For the response stimulus pathway, GO terms for the response to the endogenous stimulus, organic substance, and hormone stimulus were enriched. The analysis also showed that plants may have induced the salvage pathway in response to cold stress *via* the enriched GO terms such nucleotide salvage, purine nucleotide salvage and IMP salvage (Fig. 5a & Additional file 1: Table S11). Although most of differentially expressed miRNAs are involved in stress responses, some of them may also affect ion transport, such as the presence of the enriched GO term sulphate transport. Regarding the molecular function category, the transcript regulation activity pathway was enriched, such as the DNA binding, ADP binding, and transcript factor activity GO terms. The GO terms related to the sulphate transmembrane transport activity were both enriched in molecular function and biological process (Fig. 5b & Additional file 1: Table S11). For the cellular component category, the membrane-bounded organelle, intracellular membrane-bounded organelle, and nucleus GO terms were enriched, suggesting that the membrane component was affected under cold stress, leading to the change in membrane permeability that may influence ion transport. Interestingly, most response genes were found to be located in the nucleus (Fig. 5c & Additional file 1: Table S11). Overall, our work showed that differentially expressed miRNAs may respond to cold stress by regulating their target genes.

## Discussion

**Fig. 6:**
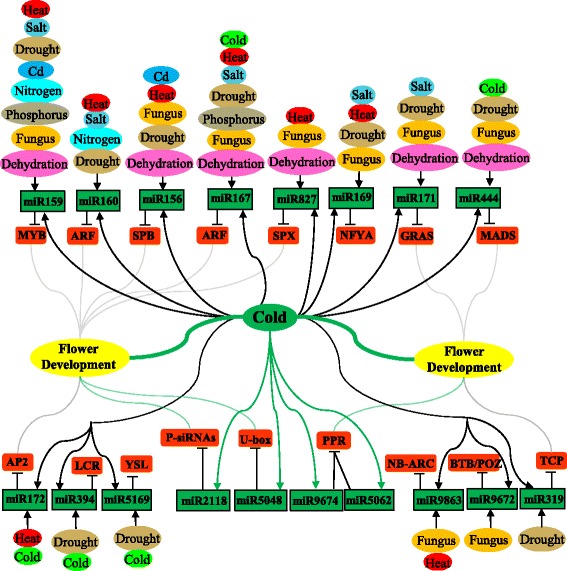
The interactive pathways of stress-responsive miRNAs and their targets. The cold-stressed microRNAs include information summarized from Xin et al (chartreuse oval) and this study (forest green oval). The forest green link indicated the unique regulation between the miRNAs and targets in response to cold stress only in our study, and the dark blue link indicated the target genes involved in the flower development pathway

MiRNAs demonstrate important regulatory functions in adaptive responses to biotic and abiotic stresses in plants [9, 11, 12, 43]. Budak and colleagues had summarized the stress-associated miRNAs and their related regulatory mechanisms in cereals and *Triticum* [9–11, 43]. MiRNAs as the post-transcript regulators, had been detected as multi-responsive to multi-environmental conditions individually and/or together with their various miRNA partners [9, 11]. Some of miRNA behaved the regulation roles with the great potential for the stress responses such as drought stress in the wild emmer wheat [64]. In *Triticum*, several studies have shown miRNAs responding to abiotic stresses such as salt, phosphorous, drought, and heat stress, and biotic stresses such as powdery mildew and stripe rust infection [27, 28, 31, 32, 43, 64, 65]. Here, we compared cold responding miRNAs with those responsive to various other stresses and developmental conditions and present an interactive analysis of miRNAs in multiple regulatory pathways, which resulted from the high-through sequencing of small RNAs (Fig. 6).

### MiRNAs are extensively involved in cold responses in plants

The sensitive response of miRNA expression levels during cold treatment indicates that miRNAs are important regulatory nodes for plant cold response. Although different species confer different set of miRNAs responding to cold, there a set of core miRNAs that are shared by most species. In wheat, 39 miRNAs from 28 families were differentially expressed under cold treatment. Among them, 12 (31%) were conserved in plants, *i.e.,* conserved in *Arabidopsis*, *populus*, *Brachypodium*, and *Medicago* [41]. The remaining appears to be temperate grass-specific. It is not clear if plants growing in cold weather develop more complicated cold response mechanisms. Judging from the functions of their targets, we found that genes regulated by cold are often involved in other stress response such as heat, drought, and salt stress. Many conserved miRNAs are also closely involved in plant development. Despite this, some miRNAs seem to be functioning mainly in stress responses. For instance, MiR394 is a cold response miRNA in wheat as shown in this study. MiR394 targets the *LEAF CURLING RESPONSIVENESS* (*LCR*) gene which is involved in salt and drought stress responses, and Fe-responsive in *Arabidopsis* [9, 66]. Similar, miR394 was found to respond to drought stress in wheat as well [29], while grass-specific miRNA miR5169 targets *YELLOW STRIPE LIKE 6* (*YSL6*) which together with *YSL4* controls iron release from chloroplasts [67]. The differential expression of miR5169 under cold stress may affect the iron transporter as suggested by Gene Ontology analysis. In addition, miR169 was identified to respond to cold stress by the regulation of *NFYA* in *Arabidopsis* [12, 13], *Populus* [15], and *Brachypodium* [16]. MiR169 as a big miRNA family in plants were detected with various expression in different tissues [9, 42], which also response to drought, salinity, heat and powdery mildew resistance [11, 43]. However, only two miRNA members miR169g and miR169n(o) with the drought responsive element and ABA-responsive element were showed to the response to the drought and salinity stresses through ABA-dependent and ABA-independent stress-inducible gene expression pathways [11]. Therefore, the post-transcriptional regulatory mode of miRNAs to their targets probably provides more efficient regulation on multiple genes that function in different biological processes.

### Co-induction of miRNAs by cold and other stresses

Although cold response miRNAs seem to be prevalent in plants, they are also responsive to other stress conditions. Up to date, most cold-responsive miRNAs also respond to additional abiotic and biotic stresses [9]. In wheat, for example, miR160 and miR167 are involved in the response to multiple biotic and abiotic stresses [27, 28, 31, 32, 34, 65]. Both miR160 and miR167 targets *auxin-response factors* (*ARFs*), but they displayed opposite expression patterns. MiR160 was up-regulated and miR167 down-regulated in this study, which is similar to their expression profiles in *Medicago* in response to cold and freezing stresses [45]. In addition, they were both heat-responsive miRNAs [9, 11]. In wheat and *Aegilops tauschii*, miR167 exhibited the reverse responses to drought stress. MiR167 is up regulated after drought stress in *Aegilops tauschii*, while down regulated in wheat [68]. For the nutrient stresses, miR160 had a role in the N homeostasis [9], and miR167 was observed to responsive to Pi deprivation in wheat [9]. In the biotic stress, miR167 were also involved in the defence responses to the infection of *Puccinia striiformis f. sp. Tritici* in wheat [43], while miR160 were responsive to the *Magnaporthe oryzae* infection in rice [11]. Thus, miRNA may regulate different auxin regulation factors involved in different stress response pathways. In wheat, miR159 is inducible by abiotic stresses, including heat, cold in this study, salt, drought, dehydration and nutrient stress such as N deficiency, Cd Stress, and phosphorus stresses, as well as biotic stress such as powdery mildew infection and *Puccinia striiformis* stress [9, 11, 27, 28, 30–32, 34, 43, 65, 69], whereas it showed transient or mild regulation under cold stress in *Arabidopsis* [13]. Differential expression of the MYB-miR159 module was detected as Aluminum toxicity-responsive in soybean and Fe-responsive in *Arabidopsis* [9]. Since over-expression of miR159 leads to male sterility, cold stress may be one important factor to inhibit anther development *via* miRNAs [70], which could be as promising candidates to be used in development of higher yielding in wheat [9, 11].

The correlation of cold responsive miRNAs with biotic response was exemplified by miR9672 which targets *BTB/POB*, a disease-resistance domain protein that is involved in the immune system responses, including those to biotic stress such as powdery mildew infection [32, 55]. Similarly, miR9863 targets disease resistance with the classical domain *NB-ARC* (Nucleotide-Binding, Apaf-1, R-proteins, and CED-4) that triggers isolate-specific immune responses against the powdery mildew fungus in *Triticum* [54]. The cross response of miRNA to biotic and abiotic stress was also observed. MiR827, for example, responds to powdery mildew infection, heat, dehydration and cold stress *via* targeting the *SPX* family [30, 32]. The *SPX* family is associated with sensitivity to cold stress in rice seedlings [71] and semi-male sterility, resulting in the reduction of grain yield in rice [72]. These versatile regulatory capabilities demonstrate miRNAs as plural potent regulator for plant stress responses.

### Development related miRNAs are sensitive to cold

Among cold responsive miRNAs, miR156 and miR172 are most important developing related miRNAs. In *Arabidopsis*, the interaction of miR156 and miR172 controls developmental timing by the regulation of *SPB* and *AP2* in *Arabidopsis* [48]. Furthermore, miR156 acts in concert to secure male fertility by targeting the *SPB* transcript factors in *Arabidopsis* [73]. Thus, overexpressed miRNA156 will increase the biomass in cereals such as the energy crop switchgrass. In wheat, miR156 responds to heat, drought, dehydration, and Cd stresses, and fungus stresses in wheat [28, 30, 32, 64], while Tae-miR172 expresses in the tapetum and microsporocytes at the anther development stage [34]. The response of miR156 and miR172 to cold, heat, and drought are conserved in *Arabidopsis*, rice, and *Brachypodium* [13, 74]. MiR171 responds to powdery mildew infection [32], dehydration [30], drought [29, 64], and salinity stresses [9] in wheat, which is also shown responding to cold stress in this study. It targeted the *GRAS* (*GAI, RGA, and SCR*) family. In GRAS, the member of GS6, plays an important role in the control of grain development in rice [75]. MiR319, which responds to both cold stress and drought stress in wheat [29], was also characterized in *Arabidopsis and* rice for cold stress [12–14, 76]. It targets members of the *TCP* gene family, which is specifically expressed in the flower organs [77], which will contribute to the higher yield in crops under stress conditions. MiR444 regulates the *MADs-box* genes, especially in monocot species, and has been demonstrated to regulate flower development [78]. In wheat, it was observed to respond to powdery mildew infection [32], dehydration [30], drought [29] and cold stress in our study. MADS28 was demonstrated to specifically regulate the floral organ number, filament length and pollen release in soybean [79]. Other less conserved miRNAs that respond to cold treatment are also involved in developmental processes. For instance, miR2118 triggers the generation of phased siRNAs, which are involved in panicle development [80, 81], while miR5062 and miR9674 targets the *PPR* protein gene, which regulates inflorescence branch development in rice [82] and can restore fertility in the cytoplasmic male-sterile line in *Brassica napus* [83]. MiR5048 targets the *U-box*. The *U-Box*/*ARM* was discovered to regulate flowering time in *Arabidopsis* [84]. Therefore, there are a number of targets of cold responsive miRNAs that also work in plant development, especially flower development, male sterility, and floret and spike development. Such an observation is interesting and may be the result of the tissue and treatment we sued in this study because the real cold tolerance genes such as *CBF*, *FR2*, *COR*, and *LEA* seem not to be the target of any miRNAs. In other words, the dramatic change in miRNA expression levels in wheat young inflorescences will cause sensitive reaction of their targets which will regulate proper flower organ development and hence the correct timing in the wild. Such mechanisms may be explored to manipulate genetically to enhance cold tolerance in wheat contributing to yield increase.

## Conclusions

This work provides the first small RNA expression profiles of young spikes at the differentiation stage of the pistil and stamen in common wheat. Our small RNA sequencing data and degradome dataset revealed regulatory roles of miRNAs during wheat cold response. The cross-response of miRNAs to multi-biotic and abiotic stresses indicated that wheat has evolved sophisticated miRNA-mediated pathways to cope with ever changing environments. Further study of these mechanisms should help our understanding of plant cold response and further secure grain yield in wheat.

## Additional files

Additional file 1
**Table S1.** Nucleotide sequences of primers for qRT-PCR of miRNAs and their targets. **Table S2.** Small RNA statistics and genome mapping information referred to the Chinese Spring genome. **Table S3.** Expression level of miRNAs in the control and cold-treated libraries. **Table S4.** Novel miRNA information, including mature, star, and precursor sequences, referred to the Chinese Spring genome. **Table S5.** Fold change of differentially expressed miRNAs and their targets by qRT-PCR. **Table S6.** Overlapped miRNAs in response to cold stress among wheat, *Brachypodium*, *Medicago*, *Populus*, and *Arabidopsis*. **Table S7.** Predicted targets of conserved miRNAs by the TargetFinder program. **Table S8.** Predicted targets of novel miRNAs by the TargetFinder program. **Table S9.** Targets of known miRNAs validated by degradome sequencing in the control and cold stress libraries. **Table S10.** Targets of novel miRNAs validated by degradome sequencing in the control and cold stress libraries. **Table S11.** Gene Ontology enrichment, including biological process, molecular function, and cellular components for the target genes of differentially expressed miRNAs after cold stress, referred as Chinese Spring. (ZIP 643 kb)
Additional file 2
**Figure S1.** Percentage of small RNAs mapped to the exon regions in control and cold stress libraries. White, grey and black rectangles represent the subgenome A, subgenome B, and subgenome D, respectively. **Figure S2.** Length distribution of tags (a) and distribution for the number of transcripts in the categories of cleavage (b) for the degradome sequencing between the control and cold stress libraries. (ZIP 10 kb)

## Abbreviations

AP2: AP2-like ethylene-responsive transcription factor
ARF: Auxin response factor
GO: Gene ontology
GRAS: GAINSENSITIVE (GAI), REPRESSOR OF gal-3 (RGA), and SCARECROW (SCR)
LRR: Leucine-rich repeat receptor-like protein kinase family
MADS: MCM1, AG, DEFA and SRF
MiRNA: MicroRNAs
NB-ARC: Nucleotide-binding, Apaf-1, R-proteins, and CED-4
NFYA: Nuclear transcription factor Y subunit A
PPR: PentatricoPeptide repeat
qRT-PCR: Quantitative real time polymerase chain reaction
SPB: Squamosa promoter binding like protein
SPX: SYG1, Pho81 and XPR1
TCP: TEOSINTE BRANCHED, Cycloidea and PCF
TGMS: Thermosensitive genic male sterile
TPM: Transcripts per million
YSL6: YELLOW STRIPE LIKE 6
