# Supplementary figures and images for: Response of microRNAs to cold treatment in the young spikes of common wheat
Source: BMC Genomics. 2017 Feb 28;18:212. doi: 10.1186/s12864-017-3556-2 (PMC5330121; doi:10.1186/s12864-017-3556-2)

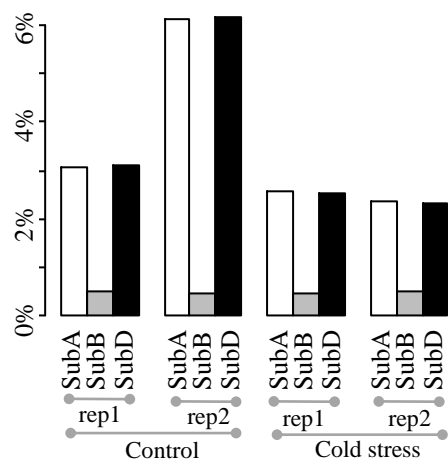

Supplement: Additional file 2 — Figure S1. Percentage of small RNAs mapped to the exon regions in control and cold stress libraries. White, grey and black rectangles represent the subgenome A, subgenome B, and subgenome D, respectively. Figure S2. Length distribution of tags (a) and distribution for the number of transcripts in the categories of cleavage (b) for the degradome sequencing between the control and cold stress libraries. (ZIP 10 kb) [file 12864_2017_3556_MOESM2_ESM.zip › Figure S1.pdf]

**(a)**

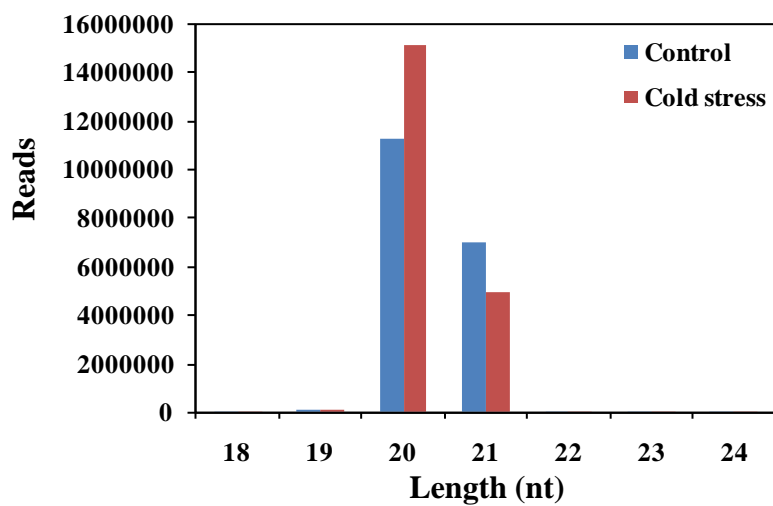

**(b)**

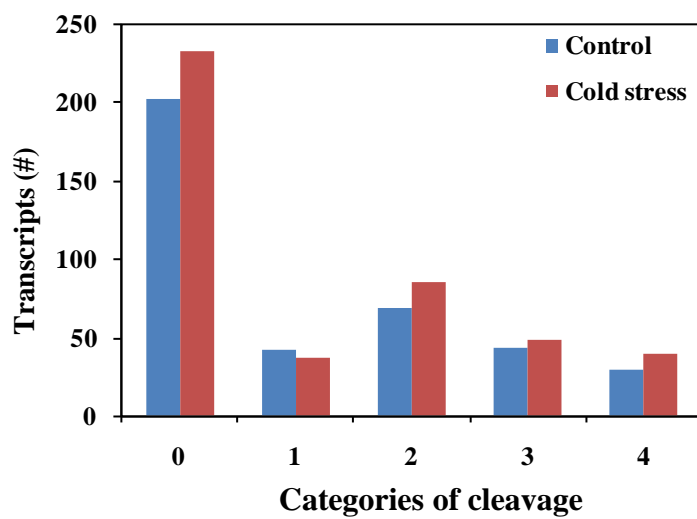

Supplement: Additional file 2 — Figure S1. Percentage of small RNAs mapped to the exon regions in control and cold stress libraries. White, grey and black rectangles represent the subgenome A, subgenome B, and subgenome D, respectively. Figure S2. Length distribution of tags (a) and distribution for the number of transcripts in the categories of cleavage (b) for the degradome sequencing between the control and cold stress libraries. (ZIP 10 kb) [file 12864_2017_3556_MOESM2_ESM.zip › Figure S2.pdf]
